# Interrelationship Amongst University Student Perceived Learning Burnout, Academic Self-Efficacy, and Teacher Emotional Support in China’s English Online Learning Context

**DOI:** 10.3389/fpsyg.2022.829193

**Published:** 2022-03-11

**Authors:** Gang Yang, Wenwen Sun, Renfeng Jiang

**Affiliations:** ^1^School of Foreign Languages and Literature, Shandong University, Jinan, China; ^2^Foreign Language School, Linyi University, Linyi, China

**Keywords:** learning burnout, academic self-efficacy, teacher emotional support, online learning context, EFL learning

## Abstract

This study seeks to explore the impact of learning burnout on university students’ English learning effect in the online environment. Through a large sample questionnaire survey, the study uses structural equation modelling to measure the interactions amongst university students’ English online learning burnout (EOLB), academic self-efficacy (AEE), and teacher emotional support (TES), thereby analysing and summarising the characteristics of their impacts on students’ online learning satisfaction. The results from the data analysis show that AEE plays a mediating role between students’ EOLB and learning satisfaction, and TES plays a moderating role between students’ EOLB and AEE, which all eventually influence students’ online learning effect manifested in aspects such as behaviour, cognition, and emotion. Given the results, the study further provides suggestions for alleviating university students’ EOLB, which can be used to optimise English online teaching design and learning practice.

## Introduction

The new postpandemic era has called for reforms in English language teaching at higher education, keeping abreast of emerging modern techniques such as online teaching and artificial intelligence, to unbind the language courses from simple physical environment. English teachers from universities thus capitalise on digital tools to devise and implement various blended teaching methods to develop students’ awareness of active learning, autonomous learning, and personalised learning. Such blended methods are well able to organically integrate the advantages of both offline classroom teaching and online intelligent learning and give full play to the supportive role of teachers and also the central position of students during the learning process. Yet, university students who are more familiar with classroom teaching may be susceptible to an array of mental weariness and low efficiency after a certain period of English online learning, for example, amotivation, distraction, lack of self-confidence, and increased anxiety, which results in what is normally defined as learning burnout. To achieve the “substantial equivalence” between offline teaching and online learning, it is of vital importance to alleviate or even eliminate this burnout through students’ English online learning.

Within the last decade or so, learning burnout has increasingly gained its popularity and becomes a new research hotspot, which prompts a wealth of literature conceived in the context of educational psychology. These studies are by and large denoted by three interrelated trends: (1) the call for cross-sectional or longitudinal research to gauge the trajectory of learning burnout of students at different levels (Law, 2007; Zhang et al., 2013; Salmela-Aro and Read, 2017), (2) the need to examine the interaction between psychological variables implicated in language learning by bringing learning burnout discussion in line with other factors such as learning motivation and learning investment (Stoeber et al., 2011; Cazan, 2015; Sulea et al., 2015), and (3) the desire to explore the mechanism of how learning burnout would affect academic performance independently or in conjunction with learning input (Salanova et al., 2010; Fiorilli et al., 2017; Paloş et al., 2019). However, these collateral perspectives are not fully recognised in the Chinese context of language teaching and learning, and very few research on learning burnout could be seen, as mainstream educational psychology at this time is yet much shaped by factors such as motivation, attitude, and anxiety. Existing studies have either analysed the macrolevel characteristics of university students’ English learning burnout and its influencing factors (Li, 2015; Wang, 2019), or at a microlevel, it discussed the respective relationships between learning burnout and learning motivation, learning behaviour (LB), and teacher–student support (Yang, 2015; Li Y. Q. et al., 2021; Wang et al., 2021). In essence, these voices conduce to channel attention closely to learning burnout in classroom settings and to the requirements of language teachers for whom the repercussion of burnout happened in virtual communication so far has few practical relevance, which, howbeit, is later disputed with the rise of online education and the development of online learning. Given that the sense of burnout in different contexts may produce dissimilar profiles and rationales (Davari et al., 2020; Li C. et al., 2021), it is necessary to revisit the issue under the online learning context and look into its manifestations from multiple views, which integrates with variables such as academic self-efficacy (AEE) and teacher emotional support (TES), to comprehensively investigate the practical actions of learning burnout on online learning and thereby optimising learning outcome. In recognition of the importance of this issue, this study seeks to use structural equation modelling (SEM) to measure and analyse the characteristics of and interactions amongst English learning burnout, AEE, and TES in online environment amongst Chinese university students, which may provide some insights into the promotion of English online learning outcome of this cohort.

## Literature

### Burnout in Foreign Language Learning

The word “burnout” is used originally referring to the negative psychological state of individuals under chronic stress, the manifestation of which on learners is often described as learning burnout (Marôco and Campos, 2012). Similar to mental syndromes such as anxiety, burnout has been traditionally seen to cause psychological, cognitive, and behavioural pressure for learners and can negatively affect the effectiveness of learning. The concept of burnout was first introduced into the research of learning in 1980s, which slightly deviated its line of enquiry from mainstream studies shaped from the beginning by pioneering psychological perspectives at that time. This shift in focus began to relate learning burnout to mental exhaustion of learners due to long-term academic pressure, gradual lack of enthusiasm for learning activities, indifference to and alienation from schoolmates, and negative attitude toward learning as a result of poor academic performance (Pines et al., 2011). Since Schaufeli et al. (2002) integrated learning burnout with the study of learning engagement, which makes it associated with studies of learner’s psychology represented by learning motivation, the learning burnout research has been moderately accepted by more educational researchers and practitioners. Leaver et al. (2005) suggested that foreign language learning would, to a certain degree, trigger learners’ anxiety, further transfer it to burnout, and called for in-depth exploration of foreign language learning burnout. Their proposal for this new concept made the burnout phenomenon closely tied to foreign language learning activities, to therefore introduced and situated burnout in the field of foreign language teaching and research. As they explained, the heavy workload of foreign language learning may cause learners’ anxiety, and burnout is the state after it reaching a certain degree (Leaver et al., 2005; Strack et al., 2015). In addition, as language learners continually evaluate and balance various internal and external motivational factors during the learning process, the degree of their engagement or effort is constantly changing. This change can be either positive or negative. The negative change of motivational state, or loss of energy, marks one of the important characteristics of burnout (Dörnyei, 2006). In other words, in foreign language learning, amotivation or demotivation to a certain level will precipitate some negative learning emotions or behaviours, including burnout (Trang and Baldauf, 2007; Dörnyei, 2009). In this sense, further researching burnout will provide a comprehensive understanding of the effect of negative emotions on learners’ foreign language learning.

Having mentioned that learning burnout serves as an important vehicle in comprehending learners’ negative emotional experience, it is used to describe exhaustion, cynicism, and reduced efficacy caused by unsatisfied learning needs, inactive learning attitude, and unqualified learning effect (Schaufeli et al., 2002). Amongst these three kernel dimensions, exhaustion (EX) refers to learners’ emotional and physical fatigue, which measures the emotion level of learning burnout. Cynicism (CY) indicates learners’ negative attitude toward specific learning tasks, and emotional and cognitive reluctance to participate in learning activities, which gauges the interpersonal dimension of learning burnout. Reduced efficacy (Ref) concerns the self-evaluation factors in learning burnout, which refers to the situation where learners feel inadequate in competency and creativity during learning process (Maslach et al., 2008; Robins et al., 2018). Based on these dimensions, the Maslach Burnout Inventory-Student Survey (MBI-SS) (Schaufeli et al., 2002) was developed and is extensively recognised and adopted to assess learning burnout amongst students. The emerging scale was later voiced independently in the Chinese context of learning and led to a number of ensuing localised learning burnout scales targeting at Chinese students (Lian et al., 2005; Wu et al., 2010). Amongst them, Wu et al.’s (2010) Adolescent Student Burnout Inventory (ASBI) has shown sound reliability and validity in all learner groups in China (elementary school, high school, and college students).

### The Mediating Effect of Academic Self-Efficacy

Academic self-efficacy is an important variable that affects and predicts students’ academic performance. It refers to individuals’ perception or belief in their abilities to complete certain academic tasks and achieve academic success, which is mainly reflected in learning ability (LA) and LB (Bandura, 2003). LA herein denotes students’ judgement of their capabilities to carry out learning tasks and attain good results, whereas LB tells students’ belief about whether they can adopt certain methods to achieve learning objectives. Based on this definition, AEE is generally perceived varying along learning satisfaction, and studies have indicated that AEE may significantly impact learning satisfaction (Aldhahi et al., 2021). For example, Shen et al. (2013) examined the relationship between self-efficacy and student satisfaction in online learning environment and found that self-efficacy predicted students’ online learning satisfaction (OLS). In exploring the self-efficacy–achievement relationship, Doménech-Betoret et al. (2017) noticed that students’ satisfaction was positively associated with AEE. Im and Lee (2021) reported that learning satisfaction was shown to have a static effect on self-efficacy perceived by a group of elementary school students. When it comes to learning burnout, Joo et al. (2015) revealed that self-efficacy and learning strategy utilisation could positively predict learning satisfaction and learning persistence, whereas learning burnout could negatively predict them. Accordingly, increasing self-efficacy and learning strategy utilisation and reducing burnout in the learning environment will improve learners’ satisfaction and their learning persistence. As a negative academic emotion, learning burnout is found to exert impacts on language learners’ self-efficacy. Charkhabi et al. (2013) used Academic Burnout Scale and General Self-Efficacy Scale to investigate the correlation between learning burnout and self-efficacy and noticed that there was a significant negative correlation between learning burnout and each dimension of self-efficacy. Kumpikaite-Valiuniene et al. (2021) studied the relationship between academic stress and learning burnout of students in four countries, namely Lithuania, Turkey, Poland, and India, respectively, using self-efficacy as moderating variable. The result corroborated the significant negative correlation between learning burnout and self-efficacy and suggested that reducing academic stress would lead to an improvement in learners’ self-efficacy, thus alleviating their learning burnout and optimising the learning effect. More recent research also indicated that AEE was a mediating factor of learning burnout on foreign language learning anxiety (Koutsimani et al., 2019). When controlling for the variable of learning burnout, there was a significant negative correlation between self-efficacy and foreign language learning anxiety. Self-efficacy played a partial mediating effect in the relationship between learning burnout and language learning anxiety, which further showed that low level of AEE was an important factor for generating learning burnout. Given the context of this study, online environment puts forward higher requirements for English learners’ LA and also corresponding LB, which is bound to produce much more complex relationship between learning burnout and AEE.

### The Moderating Effect of Teacher Emotional Support

TES is also believed to be able to affect and alleviate students’ learning burnout caused by factors such as spatial isolation and reduced interaction between teachers and students in online learning (Cleveland-Innes and Campbell, 2012). It mainly refers to teachers’ demonstration of genuine concern, understanding and respect for their students through verbal and non-verbal behaviours in the teaching process, promoting social connection and cohesion, conveying concern for students’ feelings and interest in their individuality, and honouring students’ desire to learn meaningful material and having a say in their learning. TES encompasses three dimensions: positive climate (PC), teacher sensitivity (TS), and regard for adolescent perspective (RAP). All these dimensions are regarded as pivotal in promoting students’ learning motivation (Ruzek et al., 2016). In our specific research context, TES is a two-way communicative and interactive activity based on interpersonal communication and technical media offered to enhance students’ positive emotional experience, which ultimate aim is to promote students’ autonomous learning and improve their emotional self-regulation ability (Ruzek et al., 2016; Zhao et al., 2018); that is, strengthening the online interaction between teachers and students by creating an atmosphere where teachers respect and understand students’ learning process (PC); promptly supporting students’ online learning process and meeting their emotional needs, and fostering their awareness and manners of autonomous online learning (TS); listening to students’ voices and encouraging their active thinking and inquiring (RAP).

Prior studies have explored the relationship between TES and self-efficacy, spawning inconsistent findings. Patrick et al. (2007) observed a mediating effect of math-related academic efficacy from TES on children’s task-related behaviour and learning achievement, which suggested that the effect of TES on LB and outcome may be mediated by self-efficacy. This mediating effect was somehow proved in Liu et al.’s (2018) study where the positive emotional support from teachers could promote students’ learning self-efficacy and learning engagement. Yet, inconsistency was also reported in some studies. In their examination of AEE and teacher support in relation to academic growth within one academic year, Mercer et al. (2011) found that AEE interacted with teacher support in a way that lower level of teacher support was mostly accompanied by students with high AEE. They further explained that high-achieving students usually do not require high level of perceived teacher support to attain desired academic objectives. Moreover, Kikas and Mägi (2017) looked into the relation between TES and self-efficacy in two academic skills. Interestingly, the result showed that perceptions of high TES were associated with high self-efficacy, which, in turn, influenced students’ LB and accomplishment, only in reading. Such a relation was not found between TES and self-efficacy in math or math outcome, which suggested that the result may be susceptible to the nature of different disciplinary skills.

As for the relationship between TES and learning burnout, it is reported that TES is negatively correlated with each factor underlying learning burnout and thus has negative predictive value on learning burnout (Brooks and Young, 2015). Romano et al. (2020) and Romano et al. (2021), for example, investigated the role of perceived TES in school burnout amongst a group of Italian high school students and found significant inverse effects of TES on learning burnout of this cohort. In other words, TES perceived by learners can much alleviate their burnout through learning. More specifically, amongst the three dimensions, PC has the most distinct impact on learners’ depression; TS is more likely to influence learners’ misconduct; RAP can significantly encourage learners’ sense of achievement (Zhao et al., 2018). By combing through the above literature, we are alert that the current studies on learning burnout, AEE, perceived TES, and learning satisfaction are mostly limited to univariable or two-variable studies, and there is a lack of research on the mechanism of moderation and mediation variables. Whether or how AEE will impact the relationship between students’ negative emotions such as burnout and their learning satisfaction, and further, how TES will affect this process remain unfathomed. In response to these, this study explores the mechanism by addressing how students’ perception of TES protects them from online learning burnout with the identification of some underlying processes through which students affect their learning satisfaction. Besides, the characterisation of relationships amongst these variables, to the best of our knowledge, has rarely been discussed in the foreign language learning context, particularly, in the online learning environment. The study also strives to fill this hiatus by examining the issue under the context of English online teaching and learning at Chinese tertiary institutions.

**FIGURE 1:**
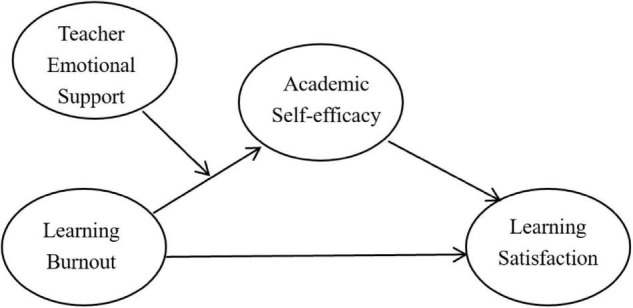
The proposed moderated mediation model.

Based on the above research motivations and literature reviewed, we propose the following theoretical model (Figure 1) and hypotheses.

H1: Learning burnout is negatively related to students’ satisfaction with online learning (H1a) and academic self-efficacy (H1b).

H2: Academic self-efficacy is positively related to students’ satisfaction with online learning (H2a) and teacher emotional support (H2b).

H3: Academic self-efficacy plays a mediating role in the relationship between learning burnout and students’ satisfaction with online learning.

H4: Teacher emotional support plays a moderating role in the relationship between learning burnout and academic self-efficacy.

## Methodology

### Research Sites, Participant, and Procedures

The sudden outbreak of current worldwide pandemic has profoundly changed the traditional patterns of teaching and learning at higher education. The pandemic hit China in January 2020, and from February, the Ministry of Education of the People’s Republic of China issued a series of policies requiring educational institutions at all levels to organise the coming teaching activities in online environment. Under such a circumstance, this study pays a selective attention to a group of graduate students’ perceptions of the online college English course. The participants contain freshmen and sophomores of non-English majors drawn from six universities in China mainland (two subordinate universities, two provincial universities of Science and Technology, and one provincial normal university). All the participating students completed the entire spring term’s (17–18 weeks, depending on academic calendar of each university) study of college English course remotely at home.

The students enrolled in the study all took a college English course, which is a basic compulsory course for undergraduate students at Chinese tertiary education. The course is designed to improve students’ comprehensive English literacy, integrating English language knowledge, practical skills, and intercultural communication as the core teaching contents. The course was organised online using various online conference platforms such as Tencent Meeting, Tencent Classroom, and DingTalk depending on teacher’s preference and technology readiness, and yet, these platforms performed very similar functions for achieving the course objectives and learning outcomes. Despite the fact that specific teaching activities and tasks varied amongst surveyed classes, all the teachers included online participation and online task completion as formative assessment to encourage students’ learning enthusiasm. Summative assessment was conducted at the end of the term, mainly consisting of a proficiency test designed to evaluate students’ all-round ability to use English.

At the end of the term (May to June 2020), we applied for and received the approval from eleven English teachers in each university, and through whom, an online questionnaire was distributed to their students. The study was also approved by the Academic Research Committee of the authors’ university. The introduction section of the questionnaire informed the participating students of the purpose and also the confidentiality of the study, and they all understood that the participation of this study was on voluntary basis and they were entitled their right to withdraw at any stage. The study obtained informed consent from all participating students. The data collection was carried out on Wenjuanxing (a Chinese online platform), and students were required to complete the questionnaire in fifteen min with consistent guidance given by the teachers.

A total of 2,054 questionnaires were distributed and collected in the preliminary round of data collection. A pilot study of the instrument was carried out amongst a group of students in Shandong University in March, 2020. We further removed 58 invalid questionnaires due to unidentifiable demographic information or fast responses (less than 3 min based on the average time used in the pilot study) and reduce the survey sample to 1,996 for analysis. Specifically, 46.1% of the surveyed students were from Humanity and Social Sciences, and the other 53.9% were from Science and Engineering. The majors included but not limited to History, Economics, Finance, International Politics, Geographic Information Science, Food Science and Engineering, Civil Engineering, Automation, and Marine Science. Also from the demographic information, the survey component involved 56.5% male students and 43.5% female students, and the proportion for freshmen and sophomores was 44.3 and 55.7%, respectively.

### Measures

Based on the definitions and dimensions of core variables in the study, Chinese university student’s English online learning burnout (EOLB) scale was worked out by synthesising a collection of scales or constructs (Appendix A). The draft of the questionnaire concerned students’ perception of learning burnout, AEE, TES, and learning satisfaction over the course of their online English learning during the COVID-19 remote learning, at the same time, assimilating technical background of online learning into the composing. To chime with the research content and objectives, elements related to the characteristics of the research scope and target such as “English learning” and “university student” were manifested in item setting and description. The final questionnaire consisted of two main sections. The first section contained items to assist the compilation of participant profile, concerning their gender, university background, major, and grade. The second section presented the university students’ EOLB scale, which included 52 items to provide an all-embracing picture of university students’ English online learning from four aspects: learning burnout (LB), TES, AEE, and OLS.

### Learning Burnout

The MBI-SS (Schaufeli et al., 2002) was frequently used in published articles to assess students’ learning burnout. Its sound factorial validity in Chinese students has been shown in Hu and Schaufeli (2009). Li C. et al. (2021) modified MBI-SS as Maslach Burnout Inventory-EFL Student Survey to fit the EFL learning context in China. In this study, the scale for the assessment of Chinese university student’s EOLB was developed from the above two surveys, which consists of 18 items within three subdimensions namely exhaustion (e.g., “I cannot fully concentrated when I study English online”), cynicism (e.g., “I do well in learning online English”), and reduced efficacy (e.g., “I doubt whether I can learn English well online”), being rated on a 7-points scale from 1 = strongly disagree to 7 = strongly agree. Confirmatory factor analysis (CFA) showed that the fit was good, and the indicators were as follows: χχ^2^/df = 3.941, RMSEA = 0.038, CFI = 0.980, TLI = 0.977, SRMR = 0.023, with factor load range from 0.708 to 0.796. Cronbach’s alpha value indicated a high internal consistency (= 0.930), KMO = 0.954. Therefore, the scale has good validity and reliability.

### Teacher Emotional Support

The TES scale was developed from Classroom Assessment Scoring System (CLASS) in Pianta et al. (2012). It was used to report the relationship between TES and behavioural engagement and also mastery motivation of adolescent students (Ruzek et al., 2016) and was adapted to explore how the perceived TES was able to alleviate Chinese university students’ burnout in online learning environment (Zhao et al., 2018). TES consists of 15 items assessing three subdimensions namely, positive climate (e.g., “The teacher creates a good English online learning atmosphere for me”), teacher sensitivity (e.g., “The teacher foresees the problems I encountered in my English online learning and takes corresponding measures”), regard for adolescent perspective (e.g., “The teacher pays enough attention to my English online learning achievements”), to be rated on a 7-points scale from 1 = strongly disagree to 7 = strongly agree. CFA indicated a good model fit, χχ^2^/df = 7.099, RMSEA = 0.055, CFI = 0.967, TLI = 0.962, SRMR = 0.023, with factor load between 0.698 and 0.813. Cronbach’s alpha value indicated a high internal consistency (= 0.922), KMO = 0.936. Therefore, the scale has good validity and reliability.

### Academic Self-Efficacy

The ASE scale was based on the Motivated Strategies for Learning Questionnaire (MSLQ) (Pintrich et al., 1993) and was validated in the EFL context by Wang et al. (2018). A total of 14 items were selected to evaluate students’ self-efficacy in LA and LB (e.g., “I think I am able to solve the problems encountered in English online learning”). Each item uses a 7-point scale ranging from 1 (complete non-conformance) to 7 (complete conformance). CFA showed that the fit was good, and the indicators were as follows: χχ^2^/df = 8.856, RMSEA = 0.063, CFI = 0.956, TLI = 0.948, SRMR = 0.031, with factor load range from 0.660 to 0.784. Cronbach’s alpha value indicated a high internal consistency (= 0.909), KMO = 0.938. Therefore, the scale has good validity and reliability.

### Learning Satisfaction

The survey on students’ learning satisfaction was adapted from studies by Wang (2003) on e-learning satisfaction, which has been extensively used in related research. The satisfaction measurement has 5 items (e.g., “I have made little progress in English online learning”), ranked on a 7-point Likert scale from 1 (strongly disagree) to 7 (strongly agree). The items describe the level of satisfaction in the online learning context by investigating students’ evaluation of the learning environment and their learning effect. A higher score indicates a higher level of satisfaction. CFA indicated a good model fit, χχ^2^/df = 17.652, RMSEA = 0.091, CFI = 0.982, TLI = 0.964, SRMR = 0.023, with factor load between 0.696 and 0.821. Cronbach’s alpha value indicated a high internal consistency (= 0.868), KMO = 0.864. Therefore, the scale has good validity and reliability.

### Data Analysis

SPSS 22.0 and Mplus 8.0 were used throughout the following procedures to analyse the data from the questionnaire. To begin with, descriptive statistics and correlation analysis were conducted to investigate basic characteristics of the students in each variable, to therefore provide the fundamental premise for framing structural model. Thence, we used Stride et al.’s (2015) Mplus code (Model 4) to identify the mediating effect of AEE in the relationship between university students EOLB and learning satisfaction, whereas bootstrapping method was adopted to estimate mediating effect and also confidence interval. Finally, we drew upon Stride et al.’s (2015) Mplus code (Model 7) to examine the moderated mediating effect of TES on university students EOLB and their AEE.

## Results and Discussion

### Construct Validity, Reliability, and Correlations

Principal component analysis (PCA) was applied in the analytical process to propose related variables, thereby extracting nine common factors with initial eigenvalues greater than 1. A cumulative variance contribution rate of 64.957% was reported from these factors, which suggested that the method was capable of incorporating key information in the dataset. The Harman single factor test (Podsakoff et al., 2003) further indicated a 27.936% variance rate of the first common factor unrotated (less than 50% standard). In other words, there was no problem with common method variance in this research context.

The result from descriptive statistical analysis of the variables (see Table 1) shows that the mean values of learning burnout dimension varied from 3.251 to 3.675, between basic disagreement and intermediate value, which indicates that the respondents found the learning burnout descriptions mostly “slightly inappropriate” or “neutral” according to their online learning experience. The mean values of remainder observed variables lie between 4.405 and 5.136, which suggests a centralised distribution of views around “slightly appropriate.” Meanwhile, to explore the reliability of the questionnaire, Cronbach’s alpha coefficients are employed to assess the internal consistency of the research construct and its dimensions, and coefficient values of 0.7 or higher are generally considered to represent an acceptable reliability (Nunnally, 1978). As seen in Table 1, the Cronbach’s alpha coefficient of each variable in this study situates between 0.855 and 0.891, which indicates high internal consistency and reliability for each variable.

**TABLE 1:** Reliabilities, descriptive statistics, and correlations of the factors.

	1	2	3	4	5	6	7	8	9
1. EX	**0.765**								
2. CY	0.537**	**0.759**							
3. Ref	0.568**	0.543**	**0.736**						
4. PC	−0.237**	−0.264**	−0.200**	**0.774**					
5. TS	−0.246**	−0.253**	−0.198**	0.562**	**0.788**				
6. RAP	−0.233**	−0.267**	−0.228**	0.583**	0.535**	**0.757**			
7. LA	−0.345**	−0.434**	−0.322**	0.268**	0.226**	0.252**	**0.725**		
8. LB	−0.355**	−0.450**	−0.334**	0.242**	0.271**	0.253**	0.540**	**0.730**	
9. OLS	−0.434**	−0.492**	−0.414**	0.307**	0.293**	0.294**	0.473**	0.428**	**0.758**
Cronbach’s alpha	0.876	0.870	0.855	0.880	0.891	0.868	0.885	0.887	0.868
M	3.251	3.585	3.675	4.849	4.946	5.136	4.405	4.538	4.583
SD	1.302	1.214	1.282	1.135	1.152	1.095	1.084	1.112	1.143
AVE	0.585	0.576	0.542	0.599	0.621	0.573	0.525	0.533	0.575
CR	0.876	0.872	0.855	0.882	0.891	0.870	0.885	0.888	0.871

*EX, exhaustion; CY, cynicism; Ref, reduced efficacy; PC, positive climate; TS, teacher sensitivity; RAP, regard for adolescent perspective; LA, learning ability; LB, learning behaviour; OLS, online learning satisfaction; AVE, average variance extracted; CR, composite reliability.*

Square root AVE is presented in the diagonal bold numbers. **P < 0.01.

Table 1 also shows the correlation coefficients of the dimensional variables in this study. From the table, there is a significant negative correlation between each dimension of the learning burnout scale and, respectively, AEE, TES, and OLS (*p* < 0.01), with the Pearson’s correlation coefficients ranging between −0.198 and −0.492. Moreover, there is a significant positive correlation between AEE and, respectively, TES and OLS (*p* < 0.01), with coefficient values in the range from 0.226 to 0.473. These achieved coefficient values indicate medium or weak correlations between the observed variables of each scale, and thus, potential high collinearity in the proposed variables is somehow avoided.

A first-order CFA model is constructed with the aid of Mplus. The results (Table 2) shows that the indices all matched the fit baseline criteria (χ^2^ = 3,270.584, df = 1,091, χ^2^/df = 2.998, RMSEA = 0.032, SRMR = 0.025, TLI = 0.959, CFI = 0.956). According to Hu and Bentler (1999), for CFI and TLI, a value >0.95 indicates a good model fit. Concerning RMSEA and SRMR, a value ≤0.06 and ≤0.08 respectively represents a good model fit. Therefore, the results indicate the measurement model is well able to fit the data. We further apply to average variance extracted (AVE) and composite reliability (CR) to evaluate the validity of the instrument. From the results (Table 1), AVE values are between 0.525 and 0.621 (greater than 0.50), and CR values are between 0.855 and 0.891 (greater than 0.70), which suggests rather good convergent validity and combined reliability amongst the latent variables (Ahmad et al., 2016). Given these analyses, reasonably sound validity is supported in the use of the construct.

**TABLE 2:** Fit index for structure equation model.

Fit	χ^2^	df	χ^2^/df	RMSEA	SRMR	TLI	CFI
Model	3,270.584	1,091	2.998	0.032	0.025	0.959	0.956
Criteria			<5	<0.08	<0.08	>0.9	>0.9

*χ^2^, the Chi-square goodness-of-fit; df, degrees of freedom; RMSEA, the root mean square error of approximation; SRMR, the standardised root mean square residuals; TLI, the Tucker–Lewis index; CFI, the comparative fit index.*

### Mediating Effect of Academic Self-Efficacy

To explore the mediating effect of AEE on the relationship between university students’ EOLB and their OLS, we used Model 4 of Stride et al.’s (2015) Mplus code to examine a mediation model for latent variable *via* Mplus 8.0, taking in learning burnout as independent variable, AEE as mediating variable, and OLS as outcome variable. The SEM results are presented as follows: χ^2^ = 233.819, df = 32, χ^2^/df = 7.307, RMSEA = 0.056, SRMR = 0.027, TLI = 0.977, and CFI = 0.967. It is noted that the chi-square/df ratio (7.307) is greater than 5 due to a large sample size in this study, and apart from that, the remainder fit indices meet the baseline criteria. In this case, it is considered that mediation model can be supported by the data, therefore demonstrating a good model fit.

**FIGURE 2:**
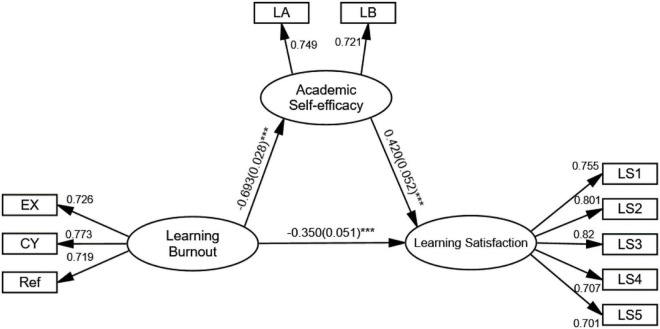
The path model for the relationship amongst university students’ English online learning burnout, academic self-efficacy, and learning satisfaction. EX, exhaustion; CY, cynicism; Ref, reduced efficacy; LA, learning ability; LB, learning behaviour; LS, learning satisfaction.

Concerning the relationship between university students’ EOLB and their satisfaction with online learning, the results (see Figure 2) reveal standardised coefficient β = −0.350 and *p* < 0.001, which means that online learning burnout has a negative impact on OLS, and therefore, H1a is supported. By the same means, learning burnout also exerts a negative effect on AEE (standardised coefficient β = −0.693, *p* < 0.001), whereas AEE is found to act a significant positive effect on OLS (standardised coefficient β = 0.420, *p* < 0.001). Given the results, H1b and H2a are supported. We subsequently estimate the standardised effect size and confidence intervals of the total effect, direct effect, and indirect effect by drawing 5,000 bootstrapped samples from the underlying data. If the confidence intervals do not contain 0, the effect can be considered as significant. As is shown in Table 3, none of the confidence intervals includes 0, and the effect size for total effect, direct effect, and indirect effect was −0.641, −0.350, and −0.291, respectively, thereby proving that all the effects are significant. After calculation, the proportion of indirect effect accounts for 45.4% of the total effect. In this regard, AEE is seen to play a mediating role, to a certain degree, between university students’ EOLB and their satisfaction with online learning. Hence, H3 is supported.

**TABLE 3:** The mediating effect test result.

	Estimate	SE	LCI	UCI	Ratio (%)
Total effect	−0.641	0.024	−0.688	−0.594	−
Direct effect	−0.350	0.051	−0.449	−0.248	54.6
Indirect effect	−0.291	0.039	−0.374	−0.220	45.4
					

The above results show that university students’ EOLB gives significant predictive power to AEE and OLS, which is in accordance with Hypothesis 1 and the findings of previous studies (Charkhabi et al., 2013; Joo et al., 2015; Kumpikaite-Valiuniene et al., 2021). Therefore, this research finding confirms that burnout is essential for students’ satisfaction and self-efficacy in their learning process (Ariani, 2017). Moreover, the results indicate that students’ AEE mediates between burnout and satisfaction in English online learning context, which confirms that AEE is the main factor affecting individual’s behavioural intention of online learning (Mheidly et al., 2020). Hypotheses 2a and 3 are verified. In other words, complexities of using digital resources and also isolation caused by remote learning may lead to a decrease in learners’ self-efficacy and will thus induce burnout-related behaviours in learning, eventually lowering their level of learning satisfaction. Given this, this study fills a gap in the literature on the mediating role of AEE between learning burnout and learning satisfaction.

In addition, a fine-grained analysis of the highest factor loading in each variable may provide a basis for further clarifying how the online learning burnout, academic efficacy, and OLS interact. To pick illustrative examples, in terms of the independent variable (university students’ EOLB), the highest loading factor is cynicism (CY) (0.773), which serves as the leading factor in online learning burnout, and is confirmed to have significant predictive power in identifying AEE and OLS, which implies that university students’ perceived self-efficacy and OLS in the online learning process are much related to their LBs and experiences (Man et al., 2019). This requires language teachers to provide immediate evaluations and feedbacks on students’ LBs over the course of learning, to foster their awareness and ability to identify and assess the English online learning method and effects, and therefore, students are able to determine whether their LB is appropriate and effective. As for the outcome variable, the factor loading of learning experience enjoyment (LEE) achieves the highest (0.820), which plays a dominant role in constructing OLS and indicates significant predictive power over both online learning burnout and AEE. This resonates with Wu’s (2016) study confirming a significant positive correlation between foreign language enjoyment and AEE, in which self-efficacy partly acts as a mediating role between foreign language enjoyment and learning engagement. In a sense, factors such as interactive and cooperative learning environment created by teachers and positive and optimistic mood possessed by students toward the English online learning process would all encourage students’ engagement in online learning and alleviate their online learning burnout, accordingly improving their self-efficacy through the process. With respect to university students’ English AEE as mediating variable, the factor loading of LA and LB is rather similar (0.749 and 0.721, respectively), both being able to notably predict online learning burnout and satisfaction, as Jovanovic et al. (2021) claim that the higher level of subjective well-being is supported with higher levels of AEE of university students. Particularly in the online learning environment, as students’ LB transforms from passive participation to active integration, their academic ability also demonstrates an obvious improvement (Wang et al., 2020), which contributes to mitigating online learning burnout and ameliorating learning satisfaction.

### Moderated Mediating Effect of Teacher Emotional Support

**FIGURE 3:**
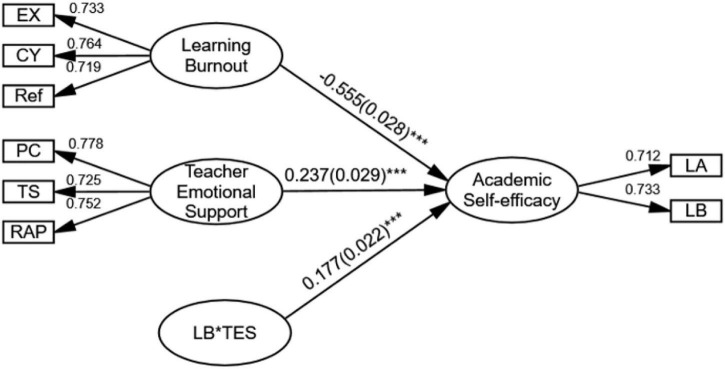
The path model for the relationship amongst teacher emotional support, university students’ English online learning burnout, and academic self-efficacy. EX, exhaustion; CY, cynicism; Ref, reduced efficacy; PC, positive climate; TS, teacher sensitivity; RAP, regard for adolescent perspective; LA, learning ability; LB, learning behaviour. ***P < 0.001.

The study also addresses the moderated mediating effect of TES on university students’ EOLB and AEE. To this end, Model 1 of Stride et al.’s (2015) Mplus code was first used *via* Mplus 8.0, which includes online learning burnout as independent variable, TES as moderating variable, and AEE as outcome variable. The results are displayed in Figure 3 (the path coefficient refers to the standardised coefficient, and standard errors are presented in parentheses).

**FIGURE 4:**
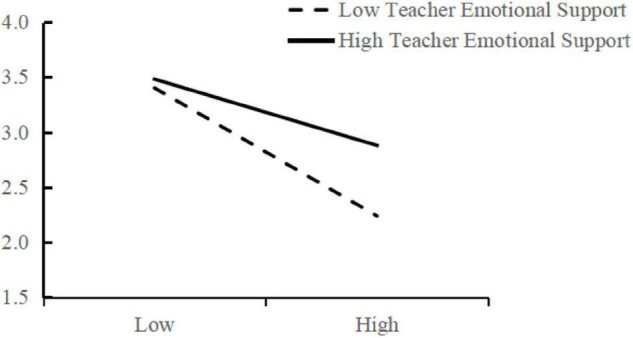
Moderating effect of teacher emotional support.

To be specific, for the interrelationship amongst the three variables, as we can see from the figure, the interpretation of TES seems to be twofold. First, the moderating variable of TES has a significant positive impact on AEE (standardised coefficient β = 0.237, *p* < 0.001). Second, concerning its moderating effect, the interaction effect LB × TES is observed to place a significant positive impact on AEE (standardised coefficient β = 0.177, *p* < 0.001). Therefore, H2b is supported. To further explain this, we picture a moderating effect diagram of TES (Figure 4) and note that the impact of learning burnout on AEE (absolute value of slope) under high TES is weaker than that under low TES. That is, TES can dilute the negative effect of university students’ EOLB placed on AEE, which means it positively moderates the influence path between learning burnout and AEE.

Based on the aforementioned results, we apply to Model 7 of Stride et al.’s (2015) Mplus code to evaluate the moderated mediating effect of TES on the relation between university students’ English learning burnout and AEE. The analytical results in Table 4 show that when the moderating variable is at low standard deviation, the estimating moderated mediating effect is −0.324, with a 95% confidence interval of −0.387 to −0.260. When the moderating variable is at high standard deviation, the estimating moderated mediating effect is −0.175, with a 95% confidence interval of −0.023 to −0.126. Furthermore, the results indicate a moderated mediation of 0.080, with a confidence interval (0.013, 0.148) (0 is not included). Accordingly, a significant moderated medicating effect of TES is disclosed on the relation between university students’ English learning burnout and their AEE. Therefore, H4 is supported.

**TABLE 4:** Moderated mediating effect of teacher emotional support.

	Estimate	SE	LCI	UCI
Low (−SD)	−0.324	0.032	−0.387	−0.260
Middle (0)	−0.249	0.027	−0.301	−0.197
High (+SD)	−0.175	0.025	−0.223	−0.126
Moderated mediation	0.080	0.034	0.013	0.148

The above analyses indicate that TES can significantly predict AEE, which is consistent with Hypothesis H2b and previous findings (Patrick et al., 2007; Liu et al., 2018). The results also illustrate that the relationship between university students’ EOLB and their self-efficacy is positively moderated by TES, which fills a gap in the literature on the relationship amongst TES, AEE, and learning burnout. Hypothesis 4 is thus verified. In the process of implementing online teaching plan, sufficient emotional support from teachers, such as promoting student autonomy, showing high expectations for student, giving continuous and clear feedback, and providing a rich of challenging, interesting, and meaningful tasks (Fredricks et al., 2016), would significantly improve students’ academic engagement and sense of achievement (Laird and Kuh, 2005; Harris and Al-Bataineh, 2016), therefore generating a positive impact on their learning effect (Yaghmour, 2016). In the meantime, student-perceived TES is able to predict students’ learning involvement, learning effort, learning strategies, learning achievement, and also their mental health (Furrer and Skinner, 2003; Sakiz et al., 2012) and can alleviate students’ learning burnout, accordingly making up for the weaknesses of online teaching such as “obvious lack of learning support and enhanced perceived loneliness” (Artino and Jones, 2012). Amongst the factors underlying TES, positive atmosphere achieves the highest (0.778), which suggests the greatest demand from the students for much dedication to establish a pleasant English online experience. In a word, TES helps to promote the in-depth interaction of students in online learning, ensure the effect of online learning in the process of deep cognitive construction (Hernandez-Selles et al., 2019), and achieve the goal of alleviating learning burnout by developing positive LB.

### Implications for Practices

The empirical data of this study reveals that university students’ EOLB, AEE, and TES are interrelated, which all can bring forth an impact on students’ satisfaction with online learning. This interrelation is characterised in our analyses as multiaction and multipathing. Amongst various paths of influence between variables, AEE plays a mediating role between university students’ EOLB and OLS, and TES acts a moderating role between online learning burnout and AEE. Both, as a result, can work on the effect of students’ online learning, manifested in many aspects such as behaviour, cognition, and emotion. Given all this, the study puts forward two suggestions to optimise online teaching design and learning practice.

First, owing to the characteristics of AEE mediating the relation between university students’ English learning burnout and OLS, university English teachers can analyse and evaluate the server and client log information of English online learning platform to explore the overall features and individual preferences of students’ online LB, through which they are able to adjust the objectives and contents of online learning tasks and identify the actual and potential development level of students, which creates the attainable zone of proximal development for them. Such a strategy will activate students’ sense of achievement during English online learning and help them to improve AEE and can eventually boost their confidence in successfully completing online learning tasks. Moreover, as online learning provides students with dynamic and individualised learning space, students are encouraged to form online community cloud to actively interact and exchange ideas with peers online. They are also recommended to take the initiative to participate in the design of online learning activities and provide feedbacks for the type, duration, and content of online activities. As the sense of tension, anxiety and burnout peter out in students’ online learning, their online learning enjoyment will gradually increase, their needs and desires for English online learning will grow, and thereby, their satisfaction with English online learning is expected to improve.

Second, as TES can moderate the relation between university students’ EOLB and AEE, university English teachers may adopt adequate measures to reinforce emotional incentives for students, which includes stimulating their motivation and enthusiasm for English online learning, developing interactive and communicative atmospheres, taking note of dynamic pattern in their learning process, and providing them with prompt and effective feedback. For example, teachers could establish digital home base as virtual classroom for students to maintain the connections with the course during the term and create individual touchpoints through the medium used in the online teaching to replace the few affectionate interactions in the classroom teaching. It is also advisable to give priorities in perceiving learning difficulties of students from internal mechanism and system behaviour in both language learning and online learning. Emotion coaching in this sense assists in recognising and coping with students’ negative emotions, and corresponding solutions such as increasing timely face-to-face communication and discussion (online video chat) will enhance their emotional experience and relieve their anxiety, fear of difficulties, and burnout. On top of that, teachers can improve the flexibility of English online teaching design and offer sufficient autonomy support for students, for instance, planning diverse language practices, encouraging students’ participation, safeguarding learning engagement, creating multidimensional interactive space, and helping students to experience a sense of accomplishment during language learning process. Giving full play to the moderating role of TES and effectively improving students’ AEE will enable students to learn to autonomously control negative emotions such as online learning burnout, so as to form teacher–student coordination mechanism to jointly promote and support English online learning.

## Conclusion

Predicated on a large-sample survey, this study constructs mediation and moderation models with latent variables to respectively explore two clusters of relations: the interrelation amongst university students’ EOLB, AEE, and OLS and the interdependence of TES, online learning burnout, and AEE. Using the proposed models and the affordances of the online learning, it is possible to sketch the characteristics of these relations and explain the influence mechanism. As our data show, online learning burnout entails a much complex system in which university students’ English LB and their mental process are interrelated and interacted with each other, which can affect OLS through the mediation of AEE and besides influence AEE moderated by TES.

This study is inevitably vulnerable to some limitations. Given that the study sample is based on students from six universities in China which may not be able to represent the entire population of Chinese university students, evidence from the study may limit its generalisability or external validity. More work is demanded to procure a more representative sample, which may give a dissimilar picture of the studied issue. Moreover, students enrolled in this study are from miscellaneous education backgrounds. Although we have controlled for potential confounding factors (college English course, online learning environment, and course assessment) most pertinent to the research aims, heterogeneous features of the participants such as language proficiency and learning strategy are assumed to vary amongst universities and majors, which may influence the results. Future research could consider further dividing the subject into different clusters or focusing on one particular homogenous group (e.g., English majors in schools of foreign languages). Finally, as the study was conducted shortly after the breakout of the pandemic when most teachers and students were still trying to accommodate to the new online environment, the result may be different when investigating the same participants in later terms or in a blended environment integrating online and classroom instruction. Future longitudinal studies could verify the research results and improve the understanding of university students’ EOLB.

## Data Availability Statement

The raw data supporting the conclusions of this article will be made available by the authors, without undue reservation.

## Ethics Statement

Ethical review and approval was not required for the study on human participants in accordance with the local legislation and institutional requirements. The patients/participants provided their online informed consent to participate in this study.

## Author Contributions

GY and RJ contributed to conception and design of the study and wrote the first draft of the manuscript. WS organised the database and performed the statistical analysis. All authors contributed to manuscript revision, read, and approved the submitted version.

## Conflict of Interest

The authors declare that the research was conducted in the absence of any commercial or financial relationships that could be construed as a potential conflict of interest.

## Publisher’s Note

All claims expressed in this article are solely those of the authors and do not necessarily represent those of their affiliated organizations, or those of the publisher, the editors and the reviewers. Any product that may be evaluated in this article, or claim that may be made by its manufacturer, is not guaranteed or endorsed by the publisher.

## Appendix

**TABLE A1:** Chinese university student’s English online learning burnout scale.

Scales	Subscales	Items	Original no.
Learning burnout	EX	I cannot fully concentrated when I study English online.	7
		I always want to sleep when I study English online.	14
		I often feel headache and eye pain in the process of English online learning.	1
		I have a low spirit at the thought of learning English online on the platform or software.	5
		I feel exhausted after completing an English online learning.	33
	CY	I do well in learning online English.	41
		I think the effect of learning English online is very good.	10
		After online learning, I am satisfied with my English improvement.	21
		I feel bored as soon as I see the English learning content on the platform or software.	15
		I can learn a lot about English during online learning.	28
		I like the challenge of English online learning.	17
		I am often at a loss facing the numerous online learning resources.	30
	rEF	I doubt whether I can learn English well online.	12
		I am disappointed that I cannot understand the English video materials on the learning platform.	20
		Compared with offline learning, learning English online freaks me out.	8
		I do not want to continue learning English online.	23
		When communicating with teachers and students online, I am not sure if my English pronunciation is accurate.	2
		I am very depressed that my English listening level has not improved after online learning.	25
Teacher emotional support	PC	I have established a friendly and harmonious relationship with my teachers in online English learning.	40
		The teacher actively participates in my English online learning activities.	26
		The teacher creates a good English online learning atmosphere for me.	34
		The teacher’s language is humorous when communicating and interacting online in English.	44
		The teacher often tells me that the effect of English online learning will get better.	6
	TS	The teacher foresees the problems I encountered in my English online learning and takes corresponding measures.	18
		The teacher inquires about my English online learning from time to time.	11
		The teacher will adjust the teaching content according to my English online learning.	27
		The teacher can provide instant responses no matter I am in a positive mood or not in English online learning.	35
		The teacher will give me individual guidance on the unique problems I have in English online learning.	38
	RAP	The teacher often gives me the opportunity to choose communicate in English online.	32
		The teacher pays enough attention to my English online learning achievements (such as oral recording or English composition submitted online).	36
		The teacher guides me to express my views or ideas in English on the learning or social platform.	3
		The teacher uses peer evaluation to promote online communication.	19
		The teacher can make an objective and fair evaluation of my English online learning.	45
Academic self-efficacy	LA	I think I am able to solve the problems encountered in English online learning.	39
		Compared with my classmate, I can find more resources to carry out English online learning.	31
		I can master English online learning content in time.	13
		I can apply what I learned online to English communication in my life.	47
		I often try to complete more difficult online English courses, even if I need to make more efforts.	42
		Even if the effect of English online learning is not positive, I can calmly analyse the problems or difficulties I encounter.	24
		No matter what I have achieved in English online learning, I never doubt my learning ability.	29
	LB	I test my English level through online communication with teachers or classmates.	43
		I know what to focus on when learning English online.	37
		When learning English online, I will take notes, such as taking down the new words.	16
		When doing English writing exercises online, I always try to recall the writing skills I learned in the classroom.	46
		I will complete the exercises online without the teacher’s requirement.	4
		I often review the English knowledge and content I have learned online.	22
		I will regularly complete online tests to check whether my English level has improved.	9
Online learning satisfaction		I think it is the right choice to learn English online on application platform or software.	48
		I have achieved the goal of English online learning.	49
		My English online learning experience is pleasant.	50
		I have made little progress in English online learning.	51
		I will not continue to study English online if I am not forced to.	52
